# Probiotics supplementation in the treatment of male infertility: A Systematic Review

**DOI:** 10.5935/1518-0557.20240013

**Published:** 2024

**Authors:** Licia Cristina Silva de Lima Oliveira, Elton Carvalho Costa, Fernanda Domingues Gomes Martins, Alcenir Sales da Rocha, Girlandia Alexandre Brasil

**Affiliations:** 1 Department of Pharmaceutical Sciences. University of Vila Velha - UVV. Vila Velha, Espírito Santo, Brazil

**Keywords:** oligoasthenoteratozoospermia, DNA fragmentation, oxidative stress, Lactobacillus, Bifidobacterium.

## Abstract

Infertility is a widespread global issue that affects approximately 15% of sexually active and active couples, which contributes to about 50% of cases. Currently, the condition remains prevalent and often inadequately treated. This systematic review aims to evaluate existing studies investigating the effects of probiotic supplementation in men. A comprehensive search was conducted across major databases, including PubMed, Cochrane, Science Direct, and Scielo, using relevant keywords such as ‘probiotic’ OR ‘Lactobacillus’ OR ‘Bifidobacterium’ AND ‘Male infertility’ OR ‘male fertility’ OR ‘sperm quality’ OR ‘sperm motility’ OR ‘oligoasthenoteratozoospermia’ and their Portuguese equivalents. Four randomized clinical studies met the inclusion criteria, focusing on men diagnosed with idiopathic male infertility (oligozoospermia, teratozoospermia, and asthenozoospermia). The findings revealed that probiotic administration exhibited promising antioxidant properties by combating reactive oxygen species (ROS), consequently protecting sperm DNA from damage that correlates with declining sperm quality. Significant improvements were observed across all sperm parameters, with notable enhancement in motility. Consequently, probiotic supplementation emerges as a potential therapeutic alternative for men diagnosed with idiopathic infertility, demonstrating positive effects on sperm quality.

## INTRODUCTION

Infertility is defined as the inability of a couple to conceive spontaneously after one year of having sexual intercourse without using any contraceptive method. Corresponding to the period that most couples get pregnant spontaneously, this does not imply, however, that the investigation of infertility is postponed until the 12-month period has elapsed (Rowe *et al.*, 1993).

This problem affects approximately 15% of sexually active couples and about 7% of men worldwide (Nieschlag *et al*., 2010). The causes of infertility are several, caused by problems in the female or male reproductive physiology. However, the male factor contributes in 50% of cases, standing out as the only cause in approximately 20 to 30% of cases (Agarwal *et al*., 2015).

In the male reproductive system, infertility is any dysfunction in the ejection and quality of semen, whether characterized by the absence, reduced quantity, or changes in sperm morphology or motility (Danis & Samplaski, 2019; Gatimel *et al*., 2017). According to Krzastek *et al*. (2020), approximately 44% of infertile men are diagnosed as idiopathic, i.e., they have no definite cause.

Moreover, Ray *et al*. (2017) point out that in the majority of male infertility cases, the abnormality is only observed after semen analysis. From sperm analysis and observation of spermatozoa, it is possible to determine whether there is a change in the number (oligozoospermia), motility (asthenozoospermia), or the presence of abnormal forms (teratozoospermia) of gametes. These abnormalities, usually present together, are called oligoasthenoteratozoospermia syndrome (OAT).

Currently, it is known that male infertility has a multifactorial origin and may result from factors such as the use of drugs and medication, endocrine and hormonal disorders, environmental pollutants, smoking, urogenital tract infections, alcohol abuse, smoking, exposure to chemical substances, among others (Brincat *et al*., 2015; Dissanayake *et al*., 2019; Mann *et al*., 2020). Despite the multifactorial feature in male infertility, significant evidence points to its strong correlation with seminal oxidative stress (Costa *et al*., 2023). Oxidative stress is a condition of seminal physiological imbalance due to an increase in reactive oxygen species (ROS) or a deficiency in total antioxidant capacity (TAC) (Pizzino *et al*., 2017).

Elevated levels of ROS can lead to a sequence of events that lead to cellular damage to sperm lipids, DNA, and proteins (Ayaz *et al*., 2018), which reduces its ability to move and fertilize. Studies show that infertile men may have elevated levels of ROS and less effective semen antioxidant capacity (Lewis *et al*., 1997; Subramanian *et al*., 2018).

Wang *et al*. (2022) point out that, despite efforts to identify the causes and develop new approaches to improve male fertility in recent decades, there are still few effective treatments to delay the decline of idiopathic male infertility. Thus, recent studies have evaluated different antioxidant supplementation strategies and their effects on ROS to improve male infertility (Bozhedomov *et al*., 2017; Kızılay & Altay, 2019; Kopets *et al*., 2020) and it has been shown that natural antioxidants or vitamin supplements, like vitamins C and E, glutathione, folate, zinc, selenium, carnitine, coenzyme Q10, lycopene, and N-acetyl cysteine, can neutralize free radicals thus improving semen parameters (Bui *et al*., 2018; Busetto *et al*., 2018; Gamidov *et al*., 2019).

However, other approaches that do not use antioxidants but can also promote antioxidant action have been gaining prominence. In this sense, the use of probiotics has grown, chiefly due to their antioxidant action and improvement in the seminal microbiota (Helli *et al*., 2022). Probiotics are defined as “live microorganisms which, when administered in adequate amounts, confer a health benefit on the host” (FAO/WHO, 2001). The most common strains belong to the genera *Lactobacillus* and *Bifidobacterium,* which are lactic acid producers and are part of many industrial and artisanal fermentations of plants, meat, and dairy products (Bolivar-Jacobo *et al*., 2023; Damián *et al*., 2022).

Microbiota describes the community of microbes that reside in almost every part of the human body. The microbiome refers to the genetic material of the microbiota, which plays roles in physiological symbiosis and pathogenic dysbiosis (Lundy *et al*., 2021). Much of the human microbiota is housed in the gastrointestinal tract, often considered an additional organ (Venneri *et al*., 2022). The dominant bacteria of the intestinal microbiota are represented by the phyla *Firmicute*s and *Bacteroidetes,* followed by the phyla *Actinobacteria*, and *Proteobacteria*, which are part of the composition of a healthy intestinal microbiota and contribute to the maintenance and protection of the intestinal epithelium (Eckburg *et al*., 2005; Rinninella *et al*., 2019).

Already in the seminal microbiome, the dominant phyla are *Proteobacteria, Firmicutes, Actinobacteria*, and *Bacteroidetes* (Yang *et al*., 2020). Lundy *et al*. (2021) found that *Prevotella* abundance was negatively associated with sperm concentration, as opposed to *Pseudomonas,* which was positively associated with motile sperm count. Additionally, increased abundance of *Ureaplasma, Enterococcus, Mycoplasma*, and *Prevotella* were negatively associated with sperm parameters. In addition, evidence shows that *Lactobacillus* can promote a protective action on semen quality, with a higher presence of this microorganism observed in samples of normal morphology (Farahani *et al*., 2021).

Thus, due to the potential of probiotics to promote the balance of the microbiome, as well as their antioxidant capacity, which has a strong association with male fertility (Wang *et al*., 2022), the objective of this systematic review is to evaluate available studies that have investigated the effects of probiotic supplementation on male infertility.

## MATERIAL AND METHODS

A systematic search of articles was conducted between February and March 2022, encompassing both English and Portuguese and without any restriction on publication date. The databases used for the search included PubMed, Cochrane (Central Register of Controlled Trials), Science Direct, and Scielo. The search terms employed were ‘Probiotic’, ‘Lactobacillus’, ‘Bifidobacterium’, ‘Male fertility’, ‘Male infertility’, ‘sperm quality’, ‘sperm motility’, and ‘Oligoasthenoteratozoospermia’. The English equivalents of these terms were combined as follows: ((probiotic) OR (Lactobacillus) OR (Bifidobacterium)) AND ((“Male infertility”) OR (“male fertility”) OR (“sperm quality”) OR (“sperm motility”) OR (oligoasthenoteratozoospermia))), with adjustments made accordingly for each database.

The articles identified underwent an initial assessment based on their titles and abstracts. Those that satisfied the selection criteria were then read in their entirety. The eligibility criteria included original published studies involving human subjects and randomized clinical trials. Studies conducted on animal models or in vitro, review articles, and duplicate studies were excluded.

The key information from the articles was entered into a spreadsheet created using the Microsoft Excel^®^ program. The extracted details included the author’s name, publication year, study design, study population, number of participants, duration of follow-up, intervention employed, and the primary findings obtained.

## RESULTS AND DISCUSSION

**Figure 1:**
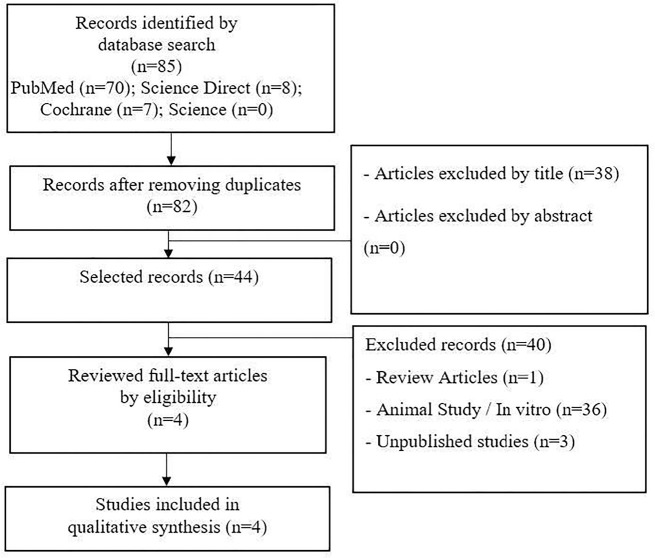
Flowchart the selection of studies for inclusion in the review.

Our review aimed to evaluate the effects of probiotic supplementation on male infertility. The systematic search in the selected databases resulted in 85 articles. After reading the titles, abstracts, and exclusions by eligibility criteria, four articles were included in this systematic review (Figure 1). Studies have shown that the use of probiotics for male infertility treatment promotes improvement in sperm parameters, such as motility, sperm concentration, morphology, semen volume, and total sperm count, thus being a relevant strategy in the therapy of male infertility.

Details of the articles included are in Table 1. The studies were published between 2017 and 2021. All were randomized clinical trials, two of which were double-blind (Helli *et al*., 2022; Maretti & Cavallini, 2017), a triple-blind (Abbasi *et al*., 2021), and a crossover study (Valcarce *et al.*, 2017). All studies were performed in men with idiopathic male infertility (oligozoospermia, teratozoospermia, and asthenozoospermia). The potential effects of probiotics have been studied in several areas of medicine. Mixtures of probiotic strains have shown to be beneficial in several outcomes, such as irritable bowel syndrome, diarrhea, atopy, respiratory tract infections, modulation of the immune system and intestinal microbiota, and inflammatory bowel disease, among others (Chapman *et al.*, 2011; La Fata *et al.*, 2018). Considering its safe use and potential therapeutic benefits, its antioxidant properties have also been gaining attention in research lines) (Mishra *et al.*, 2015; De Marco *et al.*, 2018).

**Table 1:** Description and main results of the studies included in the systematic review.

Author/Year	Design	No	follow-up time	Intervention	Results
Valcarce *et al*. (2017)	Randomized	9	6 weeks	*L. rhamnosus*CECT8361 + *B. Longum* CECT7347 (109 CFUs)	↑ sperm motility, ↓ DNA fragmentation and levels H_2_O_2_ intracellular of the sperm.
Maretti & Cavallini (2017)	Randomized, double-blind, placebo-controlled	41	24 weeks	*L. paracasei*B21060 (5 x 109 CFUs) + arabinogalactan 1243 mg + fructooligosaccharide 700 mg + L-glutamine 500 mg	↑ ejaculate volume, concentration, motility, number of spermatozoa ejaculated and percentage of typical shapes. ↑ FSH, LH and T levels.
Helli *et al*. (2022)	Randomized, double-blind	50	10 weeks	*L. casei, L. rhamnosus*, *L. bulgaricus, L. acidophilus, B. breve, B. longum*, *S. thermophiles* (2 x 1011 UFCs)	↑ Ejaculated volume, total sperm count, concentration, total motility, percentage of motile sperm. ↑ TAC and plasma MDA concentration. ↓ CRP and TNF-α. ↑ testosterone and ↓ FSH, LH and PRL, but not significant.
Abbasi *et al*. (2021)	Randomized, triple-blind	47	11.4 weeks	*L. rhamnosus, L. casei, L. bulgaricus, L. acidophilus, B. breve, B. longum, S. thermophilus*(109 CFUs) and fructooligosaccharides.	↑ sperm concentration, motility and normal morphology. ↓ lipid peroxidation and DNA fragmentation.

FSH = Follicle Stimulating Hormone; B = Bifidobacterium; E2 = estradiol; L = Lactobacillus; LH = Luteinizing hormone; MDA = Malondialdehyde; PRL = Prolactin; CRP = C-Reactive Protein; S = Streptococcus; TAC = Total Antioxidant Capacity; T = Testosterone; TNF-α = tumor necrosis factor α; CFU= Colony-forming unit.

The action mechanisms of probiotics may vary between strains and the result of a combination of events. In general, it is proposed that the main mechanisms consist of the production of antimicrobial enzymes or metabolites, inhibition of pathogenic bacteria due to competition for binding sites, competition for nutrients and modulation of the immune response (Saad *et al.*, 2013; Fan & Pedersen, 2021).

In a review study, Wang *et al.* (2017) showed that probiotic strains can exert antioxidant action in different ways, including their ability to chelate metal ions, because they have their own antioxidases enzymes, they produce antioxidant metabolites, positively regulate the host’s antioxidant enzymatic activities and negatively regulate the activities of ROS production enzymes, increase the levels of metabolites with antioxidant action in their host, regulate signaling pathways and regulate the intestinal microbiota.

In the field of female reproduction, studies have shown that the use of probiotics influences the vaginal microbiome. The benefits of probiotics as a therapy for bacterial vaginosis, increasing the abundance of *Lactobacilli*, and being compelling in preparing the vaginal microbiome before conception have been reported (Macklaim *et al*., 2015; Abbe & Mitchell, 2023; Mashatan *et al.*, 2023). Regarding male infertility, studies have shown that using probiotics improves sperm parameters and is indicated as a therapy for these patients (Maretti & Cavallini, 2017; Valcarce *et al.*, 2017; Helli *et al.*, 2022; Abbasi *et al.*, 2021).

Among the investigated parameters, we also have the lowest sperm DNA fragmentation (Valcarce *et al.*, 2017; Abbasi *et al.*, 2021), as well as the reduction of ROS levels (Valcarce *et al.*, 2017), reduced sperm lipid peroxidation (Abbasi *et al.*, 2021), reduction in malondialdehyde (MDA) levels and increase in TAC (Helli *et al.*, 2022).

### Sperm parameters

All four articles included in the present review reported improvement in sperm motility (Maretti & Cavallini, 2017; Valcarce *et al*., 2017; Helli *et al*., 2022; Abbasi *et al*., 2021). Valcarce *et al*. (2017) demonstrated a significant improvement in motility after administering a probiotic (*Lactobacillus rhamnosus* CECT8361 + *Bifidobacterium longum* CECT7347) in 9 asthenozoospermic men for six weeks. The percentage of motile sperm increased about six-fold after treatment and was sustained for six weeks after the completion of treatment.

In agreement with these results, Maretti & Cavallini (2017) reported a significant increase in progressive sperm motility (*p*<0.01) in a randomized study of 41 men with idiopathic oligoasthenoteratospermia. Supplementation was performed with probiotic Flortec®, which contained *Lactobacillus paracasei* B21060 (5 x 109 CFUs) associated with the vehicle (arabinogalactan 1243 mg + fructooligosaccharide 700 mg + L-glutamine 500 mg). In addition to the improvement in motility, other parameters were also improved, such as sperm concentration (*p*<0.01), morphology (*p*<0.01), semen volume (*p*<0.01), and total sperm count (*p*<0.01).

Following the same trend, the results found by Helli *et al*. (2022), demonstrated that daily supplementation of strains *Lactobacillus casei, Lactobacillus rhamnosus, Lactobacillus bulgaricus, Lactobacillus acidophilus, Bifidobacterium breve, Bifidobacterium longum, Streptococcus thermophiles*, (2 x 1011 CFUs), for ten weeks, significantly improved ejaculate volume (*p*=0.041), total sperm count (*p*=0.001), concentration (*p*=0.001), total motility (*p*=0.037) and the number of live sperm (*p*=0.003).

In the same way, Abbasi *et al*. (2021) noted a significant increase in motility (*p*=0.03) and improvement in sperm concentration (*p*=0.004) and morphology (*p*=0.014) parameters after supplementation with FamiLact® (*Lactobacillus rhamnosus, Lactobacillus casei, Lactobacillus, bulgaricus, Lactobacillus acidophilus, Bifidobacterium breve, Bifidobacterium longum, Streptococcus thermophilus* (109 CFU) and fructooligosaccharides) for 80 days in 47 individuals. These modifications were attributed to improvement in oxidative stress and DNA damage.

### DNA Fragmentation Index and oxidative stress

Valcarce *et al*. (2017) demonstrated a significant reduction (*p*<0.05) in the DNA Fragmentation Index (DFI) from 25.74% to 21.11% after three weeks (T1) and 21.58% after six weeks of treatment (T2), being correlated with improvement in motility. After the washout, the improvement in DFI was not sustained, increasing to 21.64% three weeks after the end of treatment (W1) and 23.09% after six weeks (W2).

Abbasi *et al*. (2021) observed a favorable improvement in DNA fragmentation (*p*=0.005) in patients who received FamiLact^®^. In the placebo group, a decrease in post-treatment DNA fragmentation was also observed, however, to a lesser extent (*p*=0.03). Concomitantly, the staining technique with A3 chromatin (CMA3) was performed, with no significant change in CMA3 positivity (*p*=0.03).

During spermatogenesis, chromatin undergoes remodeling, replacing nuclear histones with protamine, a positively charged molecule. This change enhances DNA stability and reduces damage susceptibility (Bennetts & Aitken, 2005). Abbasi *et al*. (2021) propose that the elevated protamine levels might not be the sole reason for reduced DNA fragmentation. Instead, their study supports the hypothesis that probiotics’ ability to lower ROS levels contributes to the prevention of DNA damage, thus highlighting their potential as antioxidants.

Oxidative stress was assessed by three of the four studies included in this research (Valcarce *et al*., 2017; Helli *et al*., 2022; Abbasi *et al*., 2021), demonstrating that probiotics promote antioxidant action. A study performed by Valcarce *et al*. (2017), using an *in vitro* model to analyze the antioxidant action of probiotics in *Caenorhabditis elegans*, demonstrated a higher percentage of worm survival after incubation with the strains (*L. rhamnosus* CECT8361 58.5% and *B. longum* OCECT7347 66%) of that of the positive control replicas, using vitamin C, thus confirming the antioxidant activity of the two strains.

Intracellular H2O2 levels were also analyzed to confirm that the antioxidant properties of probiotics in preventing DNA fragmentation would be a consequence of the reduction of ROS. The dichlorodihydrofluorescein assay (DCFH-DA) was used to measure H_2_O_2_ levels, with the percentage of positive cells reduced in relation to the control (16.57±3.34%) after probiotic ingestion in T1 (5.02±0.93%) and T2 (6.2±1.77%), remaining even after washout W1 (7.27±2.37%) and W2 (7.9±1.36%) (Valcarce *et al*., 2017).

Helli *et al*. (2022) evaluated serum and seminal MDA levels, a lipid oxidation product, and used a colorimetric method to analyze serum and seminal CAT. As a result, they found that TAC and MDA levels in plasma and semen in the intervention group significantly changed compared to placebo (*p*<0.001 and *p*=0.002, respectively). Additionally, Abbasi *et al*. (2021) used a BODIPY probe to evaluate sperm lipid peroxidation and found a significant reduction (29.53%±19.4 to 26%±18.82; *p*=0.02) after supplementation.

A previous *in vitro* study by Barbonetti *et al*. (2011) provided initial evidence supporting the effectiveness of probiotics in safeguarding spermatozoa against lipid peroxidation and its detrimental impact on sperm motility and viability. The study employed a combination of three *Lactobacillus* strains. In a separate study conducted on rats fed a high-fat diet, significant reductions were observed in the activities of superoxide dismutase (SOD) and glutathione peroxidase (GSH-Px) in both serum and sperm, compared to control rats. This group exhibited elevated levels of malondialdehyde (MDA) and nitric oxide (NO), indicating the presence of oxidative stress and lipid peroxidation. However, supplementation with probiotics restored SOD and GSH-Px activities and reduced MDA and NO levels, approaching those of the control group. That demonstrates the antioxidant properties of probiotics and their ability to mitigate oxidative damage to some extent. It has been established that excessive free radicals can diminish sperm count, viability, and motility (Chen *et al*., 2013).

It is known that defective spermatozoa are more vulnerable to damage caused by oxidative stress, because the composition of their plasmatic membranes contains high percentages of polyunsaturated fatty acids (PUFAs). This condition favors the generation of ROS by sperm mitochondria, inducing an increase in lipid peroxidation production process by the sperm in addition to the lack of cytoplasmic enzyme system. In addition to lacking cytoplasmic enzyme systems necessary for repairing damage induced by oxidative stress (Agarwal *et al*., 2003; Aitken *et al*., 2010; Darbandi *et al.,* 2018).

ROS (Reactive Oxygen Species) inflict initial damage to the sperm membrane, consequently impairing its motility and ability to fuse with the oocyte (Darbandi *et al*., 2018). The precise mechanisms underlying reduced sperm motility due to oxidative stress remain uncertain. However, studies suggest that it may be attributed to axonemic alterations resulting from potential depletion of intracellular adenosine triphosphate (ATP), as well as tail abnormalities leading to decreased sperm motility (Ayaz *et al*., 2018; de Lamirande & Gagnon, 1992).

Oxidative stress can also cause DNA damage, accelerating the cell apoptosis process and consequent decrease in sperm count, being correlated with infertility and decreased semen quality (Agarwal *et al*., 2003; Ayaz *et al*., 2018; Sumner *et al*., 2019).

Such evidence and results found in our review reinforce this hypothesis that the administration of probiotics exerts an antioxidant action, acting in defense against ROS and damage to sperm DNA, with consequent improvement in sperm parameters, with emphasis on motility.

### Sex hormones

Two studies (Maretti & Cavallini, 2017; Helli *et al*., 2022) evaluated blood levels of sex hormones after intervention with probiotics. Maretti & Cavallini (2017) observed that follicle-stimulating hormone (FSH), luteinizing hormone (LH), and testosterone (T) levels increased (*p*<0.01), estradiol (E2) and prolactin (PRL) levels did not increase significant changes. On the other hand, Helli *et al*. (2022) observed increased testosterone levels and decreased serum levels of FSH, LH, and PRL. However, these findings were not statistically significant (*p*=0.063, *p*=0.21 *p*=0.109, and *p*=0.128, respectively). These results, although inconclusive, suggest that the use of probiotics may influence hormonal regulation.

Hurtado de Catalfo *et al*. (2007) suggested that supplementation with antioxidants can improve the clinical picture of men with varicocele. Authors suggest that hormonal alterations may be associated with an indirect effect of ROS on the action of Leydig cells, mainly on the regulation of testosterone levels (Darbandi *et al*., 2018). Furthermore, evidence had already shown that oxidative stress is capable of inhibiting steroidogenesis in these cells (Mancini *et al*., 2023).

Thus, the hypothesis is that increased levels of testosterone and changes in gonadotropins may be related to the reduction of oxidative stress. Since the results are controversial and inconclusive, the physiological significance of the alterations needs to be clarified and the real impact of probiotics on hormonal factors evaluated.

### Inflammatory factors

Regarding inflammatory factors, these were investigated only by Helli *et al*. (2022), who determined serum levels of tumor necrosis factor α (TNFα) and C-Reactive Protein (CRP), demonstrating that after probiotic supplementation, there was a significant reduction in both CRP and TNFα (*p*=0.001 and *p*=0.003, respectively). Similarly, Chen *et al*. (2012) investigated the *in vivo* effects of the *Lactobacillus kefiranofaciens* M1 strain on the intestinal epithelial cells of mice with colitis induced by Dextran sodium sulfate (DSS), and as a result, they observed that the use of the strain significantly reduced the production of pro-inflammatory cytokines (IL-1β and TNF-α) and increased production of the anti-inflammatory cytokine (IL-10), thus suggesting the potential anti-inflammatory activity of the strain.

Nanda Kumar *et al.* (2008) in a previous study of mice treated with DSS, suggested that probiotic strains can change the profile of cytokine secretion from pro-inflammatory to anti-inflammatory. Thus, the evidence and results found in our review demonstrate the potential anti-inflammatory action of probiotics.

In addition, considerable advances in studies of the microbiota associated with the intestinal and genital tracts have been correlating microbial communities and the effects of bacterial dysbiosis on infertility (Venneri *et al*., 2022). Studies reviewed by Farahani *et al*. (2021) reported a greater abundance of *Prevotella* and *Staphylococcus* in semen, negatively correlating with motility. Abnormal sperm morphology was correlated with decreased *Lactobacillus.* Likewise, Lundy *et al*. (2021) demonstrated that the testicular microbiome may play a significant role in spermatogenesis and that intestinal and urinary dysbiosis may be a factor that influences infertility.

Monteiro *et al*. (2018) found an increased amount of *Pseudomonas, Klebsiella, Aerococcus, Actinobaculum, Neisseria* and a lower presence of *Lactobacillus* in groups with oligoastenoteratozoospermia and hyperviscosity. A lower prevalence of *Lactobacillus* and *Propionibacterium* was also observed. These are Gram-positive bacteria, which seem to affect maintaining semen quality, as well as the potential to lessen the negative influence of gram-negative bacteria like *Prevotella, Aggregatibacter*, and *Pseudomonas* (Weng *et al*., 2014).

The intestinal epithelial cell layer acts as the primary barrier for molecule permeation, allowing the passage of paracellular or transcellular molecules. Modifications in membrane composition affect this barrier, leading to changes in intestinal permeability (Ghosh *et al*., 2021). Besides safeguarding the host against infections and pathogens in the gastrointestinal tract, the intestinal barrier facilitates the proper absorption of nutrients from food and fluids (Slifer & Blikslager, 2020).

Probiotics have been found to impact intestinal pathophysiology in various ways, with compelling evidence suggesting their ability to enhance intestinal barrier function. That is achieved through the activation of genes involved in the formation of healthy tight junctions, which serve as adhesive connections between intestinal epithelial cells (La Fata *et al*., 2018). *In vitro* and *in vivo* studies conducted by Blackwood *et al*. (2017) demonstrated that specific strains of *Lactobacillus* probiotics strengthen the intestinal barrier and maintain tight junction integrity.

However, it is relevant to note that the study mentioned has certain limitations, including a limited number of available articles on the subject and small sample sizes. Despite variations in probiotic administration, composition, dosage, and duration among studies, all have shown promising results in improving sperm parameters and potential treatment of male infertility.

## CONCLUSION

The findings in our review demonstrate the benefit of probiotics treatment in male infertility. Confirming the results of improvement in sperm parameters, such as motility, sperm concentration, morphology, semen volume, and total sperm count, after treatment with probiotic strains (*Lactobacillus, Bifidobacterium,* and *Streptococcus*). It is considered a safe and affordable treatment. The still limited number of studies available on the use of probiotic therapy as an intervention in male infertility is highlighted, and it is suggested that larger-scale studies be performed to elucidate the mechanism of action and application of probiotics.
